# Chemical Recycling of PET Using Catalysts from Layered Double Hydroxides: Effect of Synthesis Method and Mg-Fe Biocompatible Metals

**DOI:** 10.3390/polym15153274

**Published:** 2023-08-02

**Authors:** Ana P. Arcanjo, Denisson O. Liborio, Santiago Arias, Florival R. Carvalho, Josivan P. Silva, Bernardo D. Ribeiro, Marcos L. Dias, Aline M. Castro, Roger Fréty, Celmy M. B. M. Barbosa, Jose Geraldo A. Pacheco

**Affiliations:** 1 Laboratory of Refining and Cleaner Technology (LabRefino-Lateclim), Department of Chemical Engineering, Institute for Petroleum and Energy Research (i-LITPEG), Federal University of Pernambuco, Recife 50740-550, PE, Brazilariashenao@gmail.com (S.A.);; 2 Fuel Laboratory, Department of Chemical Engineering, Institute for Petroleum and Energy Research (i-LITPEG), Federal University of Pernambuco, Recife 50740-550, PE, Brazil; 3 Engineering and Technology Center, Uninassau University, Paulista 53401-440, PE, Brazil; 4 Biochemical Engineering Department, School of Chemistry, Federal University of Rio de Janeiro, Rio de Janeiro 21941-909, RJ, Brazil; 5 Macromolecules Institute, Federal University of Rio de Janeiro, Rio de Janeiro 21941-598, RJ, Brazil; 6 Research, Development and Innovation Center (Cenpes), Petrobras, Rio de Janeiro 21941-915, RJ, Brazil

**Keywords:** plastic, depolymerization, glycolysis, oxide catalysts, hydrotalcite, BHET

## Abstract

The chemical recycling of poly(ethylene terephthalate) (PET) residues was performed via glycolysis with ethylene glycol (EG) over Mg-Fe and Mg-Al oxide catalysts derived from layered double hydroxides. Catalysts prepared using the high supersaturation method (h.s.c.) presented a higher surface area and larger particles, but this represented less PET conversion than those prepared by the low supersaturation method (l.s.c.). This difference was attributed to the smaller mass transfer limitations inside the (l.s.c.) catalysts. An artificial neural network model well fitted the PET conversion and bis(2-hydroxyethyl) terephthalate (BHET) yield. The influence of Fe in place of Al resulted in a higher PET conversion of the Mg-Fe-h.s.c. catalyst (~95.8%) than of Mg-Al-h.s.c. (~63%). Mg-Fe catalysts could be reused four to five times with final conversions of up to 97% with reaction conditions of EG: PET = 5:1 and catalyst: PET = 0.5%. These results confirm the Mg-Fe oxides as a biocompatible novel catalyst for the chemical recycling of PET residues to obtain non-toxic BHET for further polymerization, and use in food and beverage packaging.

## 1. Introduction

Technological advancement enables the replacement of numerous raw materials with plastic; at the same time, however, the extensive use of plastic has caused an environmental issue. Poly (ethylene terephthalate) (PET) has extensive applications resulting in a great amount of waste without proper disposal or treatment [1]. Plastics in the environment generate micro- and nanometric particles that are deposited in living organisms. Particles larger than 700 nm have been found in human blood, in a concentration of 1.6 μg mL^−1^, these being mainly of PET, polyethylene and polystyrene origin [2]. Thus, PET can be considered a potentially very hazardous material when disposed of in the environment; then, it has to be effectively recycled after use [3]. The main plastic recycling method is mechanical; this, however, requires clean material to process, without other residues and not mixed with other types of plastics. PET residues not suitable for mechanical recycling can be used to burn for energy recovery or be submitted to chemical recycling [4]. Different methods of PET chemical recycling have been studied, such as aminolysis, methanolysis, hydrolysis and glycolysis, the latter being considered economically viable [5].

Glycolysis reaction occurs through a transesterification between ester groups of the PET molecule and ethylene glycol (EG), producing lower molecular weight molecules, such as oligomers, dimers and bis(2-hydroxyethyl) terephthalate, or BHET [1]. Zinc acetate is the most effective homogeneous catalyst; in this case, however, the catalyst is lost or incorporated into the BHET product without the possibility of catalyst reuse. Heterogeneous catalysts may offer advantages such as the recovery for reuse in glycolysis and less contamination of the BHET product obtained [1]. Several heterogeneous catalysts have been reported, giving high PET conversions, such as ZnO and Mn_3_O_4_ with silica [6], ZnMn_2_O_4_ spinels [7], ionic liquids [8] and MOFs [9]. 

Various iron-based catalysts have been shown active in PET glycolysis. Examples of such catalysts include Mg-Al-O@Fe_3_O_4_ [10], cobalt–ferrite [11], iron–ionic liquid [12], magnetically recoverable γ-Fe_2_O_3_ [13], Fe_2_O_3_/N-graphene [14], Fe_3_O_4_-carbon-nanotubes [15], spinel ferrites of zinc or copper [16], Fe_2_O_3_@MoS_2_-2D nanocomposites [17], and 2D-Fe(III) nanosheets from fluid-shear exfoliation [18]. The use of catalysts such as MgO [19] and Mg-Zn-Al [20] also resulted in high conversions for PET glycolysis. The works recalled above show that Mg or Fe oxides can be used as active catalysts for PET glycolysis. However, a combination of Mg and Fe oxides, without other elements, has not been reported yet for the chemical recycling of PET. Pyroaurite is a mineral containing Mg and Fe with a rhombohedral structure that belongs to the large group of layered double hydroxides (LDHs) or hydrotalcite-like compounds [21]. Its application has been reported in the literature for different studies with satisfactory results, such as the adsorption of Sb from water [22], and mercury removal [23]. 

Although sensitive to the synthesis method, LDH generally produces oxides with high surface area [24,25] that are active for many types of reactions [26,27]. Another advantage of Mg and Fe is their biocompatibility with living organisms since these elements are endogenously present in the human body. This allows their use in the production of materials that can be used for packing food, although there may be traces of Mg and Fe in their composition [28]. 

Few catalysts reported for PET glycolysis have used biocompatible elements in their composition [1]. The biocompatibility of a material or an element is the quality of not having toxic or injurious effects on biological systems [29]. In this context, the oxides of Mg [30,31] and Fe [32,33,34] are biocompatible compounds, but Al is not. The study proposed in this work evaluated the influence of the synthesis method and the use of biocompatible Fe in a Mg-Fe oxide instead of Al in a Mg-Al oxide, prepared from LDH. Their morphological and textural properties as catalysts were evaluated related to the glycolysis of post-consumer PET in a chemical recycling process. The synthesis methods used in this work are related to coprecipitation at low supersaturation (at controlled pH~10) and high supersaturation without pH control. The PET glycolysis parameters established in this study were defined based on the literature review [35].

## 2. Materials and Methods

### 2.1. Synthesis of Mg-Fe Catalysts

Reagents used were Mg(NO_3_)_2_·6H_2_O (>98%, Vetec, Duque de Caxias, RJ, Brazil), Fe(NO_3_)_3_·9H_2_O (>98%, Dinâmica, Indaiatuba, SP, Brazil), Al(NO_3_)_3_·9H_2_O (>98%, Dynamics), NaOH (99.0%, Merk, Kenilworth, NJ, USA) and Na_2_CO_3_ (99.5%, Vetec, Duque de Caxias, RJ, Brazil) previously dried in a desiccator. The catalysts synthesized had the LDH as a precursor by coprecipitation methods: high supersaturation (h.s.) without pH control [36] or low supersaturation (l.s.) with controlled pH at 10 [37]. The LDH followed the formula Mg_0.75_Fe_0.25_(OH)_2_(CO_3_^2−^)_0.125_·mH_2_O; x = 0.25 was the molar ratio [Fe/(Mg+Fe)]. For comparison purposes, the Mg-Al LDH was synthesized in a similar way. For Mg-Fe low supersaturation LDH, 150 mL of the solution was prepared with (Mg^2+^~0.25 mol·L^−1^) and (Fe^3+^~0.083 mol·L^−1^) nitrates (solution I). Solution II (150 mL) used (NaOH~0.73 mol·L^−1^) and (Na_2_CO_3_~0.046 mol·L^−1^), to which were added an excess of 10% of the stoichiometric amount in the general formula to guarantee the presence of hydroxyls and compensation anions, respectively. Solution I and solution II were dripped at a 2.5 mL min^−1^ flow rate into a flask containing 400 mL of deionized water under stirring (800 rpm), maintaining the pH constant at ~10. The precipitates were aged for 3 h at 50 °C plus 18 h at 27 °C. The solids were vacuum filtered, washed with deionized water previously boiled at 100 °C, to remove dissolved CO_2_ until a neutral pH was obtained, and dried. Mg-Al LDH was prepared by replacing the Fe(NO_3_)_3_ with the Al(NO_3_)_3_ (1.67 mol·L^−1^). The synthesis of the high supersaturation Mg-Fe and Mg-Al LDH was performed with 250 mL of solution I containing (Mg^2+^~0.15 mol·L^−1^) and (Fe^3+^~0.05 mol·L^−1^) nitrates, and solution II (250 mL) prepared with (NaOH~0.44 mol·L^−1^) and (Na_2_CO_3_~0.0275 mol·L^−1^), to which was added an excess of 10% of the stoichiometric amount in the general formula to guarantee the presence of hydroxyls and compensation anions. Solution I was added by dripping in the solution II in ten minutes with no pH control. A similar method was performed to obtain Mg-Al with the same concentrations but replacing Fe for Al. The other steps were similar to the low supersaturation method. The LDH materials obtained were denoted as (Mg-Fe-l.s. and Mg-Al-l.s.) for the low supersaturation synthesis method and (Mg-Fe-h.s. and Mg-Al-h.s.) for the high supersaturation method. After calcination of these LDH materials (for 3 h at 400 °C, 10 °C·min^−1^), the corresponding oxide catalysts obtained were denoted respectively as (Mg-Fe-l.s.c. and Mg-Al-l.s.c.) and (Mg-Fe-h.s.c. and Mg-Al-h.s.c.). These oxides were ground and sieved between 88 and 125 μm.

### 2.2. PET Glycolysis and Catalyst Reuse

Bottle-grade PET waste (post-consumer) was washed and ground to 65-mesh particle size. A 250 mL reactor was loaded with 5 g PET, EG with a mass ratio EG:PET = 5:1 and catalyst: PET = 0.5%. The top of the reactor was connected to a condenser using a 15 °C circulating cold bath. The glycolysis occurred at 200 °C, under the reflux of EG. After the reaction, a hot vacuum filtration separated the catalyst and the unconverted PET. Then, 100 mL of deionized water previously boiled was added to promote the precipitation of the oligomer which was recovered by a second filtration. The liquid fraction was frozen and the crystallized BHET monomer was recovered in the third filtration [35,38]. PET conversion and BHET molar yield were obtained (Equations (1) and (2)). After each reaction, the catalyst was calcinated at 400 °C (at 10 °C min^−1^) for 60 min before being reused.

(1) $$\mathrm{PET}\;\mathrm{Conversion}\;(\%)=\frac{m_{\mathrm{PET},i}-m_{\mathrm{PET},f}}{m_{\mathrm{PET},i}}\;*\;100$$

(2) $$\mathrm{BHET}\;\mathrm{yield}\;(\%)=\left[\frac{\left(\frac{m_{\mathrm{BHET}}}{{\mathrm{MM}}_{\mathrm{BHET}}}\right)}{\begin{array}{c} \left(\frac{m_{\mathrm{PET},i}}{{\mathrm{MM}}_{\mathrm{PET}}}\right) \end{array}}\right]*\;100$$

where m_BHET_ is BHET mass, MM_BHET_ is BHET molar mass (254 g mol^−1^), m_PET,i_ is the initial PET mass, m_PET,f_ is the unconverted PET mass and MMPET is PET molar mass of the basic PET unit (192 g mol^−1^) [39,40]. BHET selectivities were calculated from (Equations (3) and (4)); m_oli_ is the oligomer mass; m_mon_ is the BHET monomer mass [41].

(3) $$S_{\mathrm{oligomer}\;\left(\%\right)=\left[\frac{m_{\mathrm{oli}}}{\left(m_{\mathrm{oli}}+m_{\mathrm{mon}}\right)}\right]*100}$$

(4) $$S_{\mathrm{monomer}\;\left(\%\right)=\left[\frac{m_{\mathrm{mon}}}{\left(m_{\mathrm{oli}}+m_{\mathrm{mon}}\right)}\right]*100}$$

The reuse tests were conducted to evaluate the catalyst’s performance over multiple cycles. After each glycolysis reaction, the catalyst was recovered by vacuum filtration and subjected to a drying period at 70 °C for 24 h. Subsequently, it was calcined at 400 °C (10 °C·min^−1^) to ensure the removal of any remaining PET fragments. The resulting catalyst was then utilized in the next glycolysis cycle.

### 2.3. Characterization of Mg-Fe Materials and BHET Products

XRD patterns were obtained from a Shimadzu-XRD-6000 diffractometer (Kyoto, Japan) using Cu-K (λ = 1.54056 Å), 30 kV, 30 mA, 0.02° step size, at 2° min^−1^, 2θ~5–70°. FTIR spectra were obtained on a Bruker-Tensor-27 spectrometer (Billerica, MA, USA) with ATR, range 4000–800 cm^−1^, 30 scans. The N_2_ adsorption/desorption was performed on a Micrometrics-ASAP-2420 apparatus (Ottawa, ON, Canada). Metal composition was obtained on an EDX Rigaku-NEX-DE spectrometer (Tokyo, Japan), 100 s irradiation time under helium. SEM images were captured using a Mira3-Tescan apparatus, 10 keV, 15 nm WD, SE-detector. Thermogravimetric-TG and differential scanning calorimetry-DSC analyses were performed on a NETZSCH-STA-449-F3 instrument (Selb, Germany), using 5–10 mg sample, 30–800 °C at 10 °C min^−1^ under 20 mL·min^−1^ of air for catalysts and nitrogen for BHET. The purity of BHET was determined from (^1^H-NMR) on a 300 MHz Agilent-VNMRS300 device (Santa Clara, CA, USA), with samples dissolved in CDCl_3_.

### 2.4. PET Glycolysis Kinetics and Neural Network Modeling

The kinetic evaluation of PET glycolysis was conducted by assessing the reaction at different times: 10, 20, 30, 40, 50, 60 and 70 min. After each elapsed time, the reaction system was stopped and removed from the heating mantle, and the filtration and recovery procedures immediately proceeded. These experiments were performed in duplicate.

The PET glycolysis pseudo-homogeneous kinetic model is given in (Equation (5)), using [EG] and [PET] concentrations.

(5) $$-\frac{d\left[\mathrm{PET}\right]}{\mathrm{dt}}=-k^{\prime }\left[\mathrm{EG}\right]\left[\mathrm{PET}\right]$$

Because EG concentration is in excess, it can be considered constant [41,42]. Thus, the model can be written in terms of PET conversion “x” in integrated form (Equation (6)).

(6) $$\ln\left(\frac{1}{1-x}\right)=k\;t$$

A neural network model (ANN) was obtained using the results from experimental data to predict the concentrations of PET, BHET-oligomer and BHET-monomer as the output variables, as a function of reaction time (input variable). Experimental data were normalized in the range [–1, 1] and used to estimate the parameters of a feed-forward ANN model with a sigmoidal activation function, using C# language in Unity 3D© 2019.3.5f [43,44]. The ANN learning method used was adapted from the random search optimization method and genetic algorithm previously described [44], aiming to minimize the absolute mean error.

## 3. Results

### 3.1. Characterization of the Catalyst

**Figure 1:**
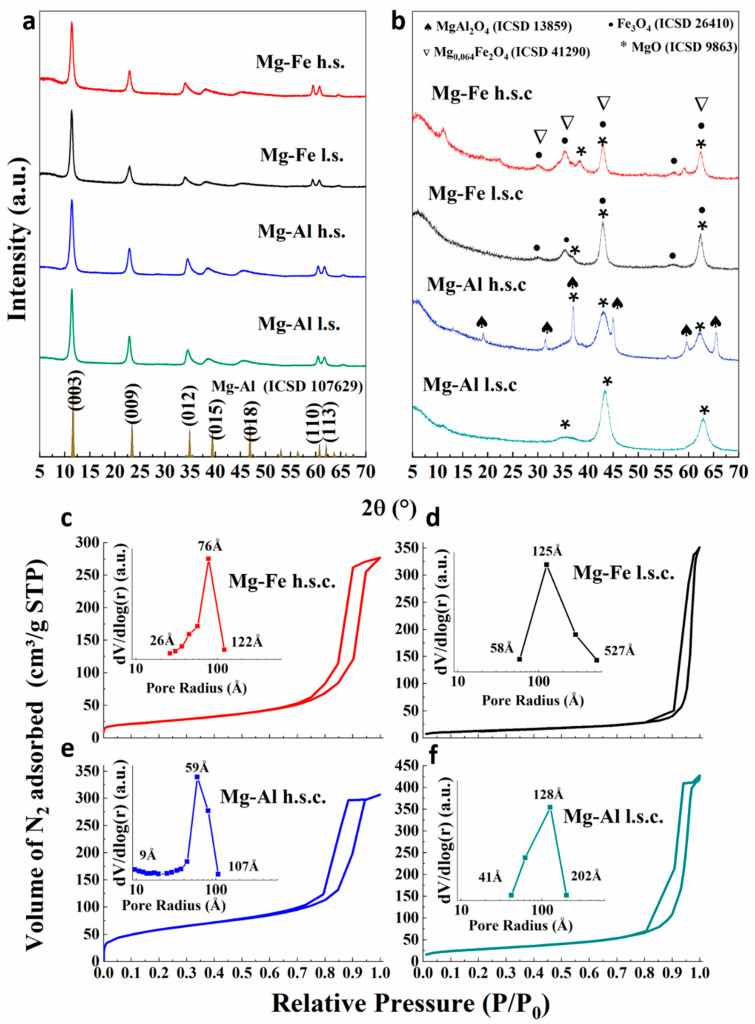
(**a**) XRD patterns of Mg-Al and Mg-Fe LDHs; (**b**) XRD of the oxides; (**c**–**f**) N2 adsorption–desorption isotherms and pore size distribution of the Mg-Fe and Mg-Al mixed oxides.

Figure 1a shows diffractograms with well-crystallized LDH phases of Mg-Al-CO_3_ hydrotalcite (ICSD 81963), with reflections and crystalline basal planes at 11° (003), 23° (006) and 34° (009), and asymmetric reflections with 2θ angles and non-basal planes at 35° (012), 39° (015), 47° (018), 60° (110) and 61° (113). The calculated unit cell parameters (Table S1, Supplementary Materials) were “*a*”, related to the distance between two cations, and “*c*”, equal to the triple of the basal distancing (3d) (Equations (7)–(9)).

(7) n.λ=2d.senθ

*a* = 2*d* (110) (8)

*c* = 3*d* (003) (9)

Cell parameters were close to the pyroaurite Mg_6_Fe_2_(OH)_16_CO_3_·4.5H_2_O (*a* = 3.109 Å; *c* = 23.41 Å) [45] and hydrotalcite Mg_6_Al_2_(OH)_16_CO_3_·4H_2_O (*a* = 3.054 Å; *c* = 22.81 Å) [46] phases with polytype 3R, respectively for the Mg-Fe and Mg-Al samples [47]. The larger “*a*” value for Mg-Fe was due to the larger ion radius of Fe^3+^ (0.64 Å) compared to Al^3+^ (0.50 Å). 

Figure S1 shows that Mg-Al LDHs were more crystalline than Mg-Fe LDHs, because the incorporation of Fe^3+^ cations with higher ion radius than Mg^2+^ can introduce distortions in the layers, negatively influencing the ordering of the material [48]. Crystallite sizes of the LDHs were calculated using Scherrer’s equation to the most crystalline reflection (003-plane) (Table S1). The materials synthesized using the low supersaturation method showed a larger crystallite size than those using the high supersaturation method. The high supersaturation condition favored fast precipitation during the synthesis, resulting in materials with more compact structures. On the other hand, coprecipitation at low supersaturation condition promotes the slow growth of larger and more crystalline crystals [49].

XRD patterns of calcined catalysts are shown in Figure 1b. The oxides obtained from the low supersaturation method (l.s.c.) were more crystalline than those from the high supersaturation method (h.s.c.). Reflection peaks from l.s.c. oxides presented greater relative intensity and smaller width. This agrees with the results of the LDH precursors presented in Figure 1a. In all oxides, the MgO phase (ICSD 9863) was identified. For the Fe-materials, a slight crystallization of the magnetite phase Fe_3_O_4_ was identified (ICSD 26410). The formation of a Mg_0.64_Fe_2_O_4_ spinel phase (ICSD 41290) was observed in the Mg-Fe-h.s.c. and MgAl_2_O_4_ (ICSD 13859) in the Mg-Al-h.s.c. catalyst.

Figure S2 shows that the TG-DTG curves presented typical mass losses of LDH materials including dehydration, dehydroxylation and decarboxylation [27]. Table S2 shows the mass losses of each temperature range, emphasizing the differences between LDH synthesis methods and composition [50]. The FTIR spectra of LDH materials, plotted in Figure S3, show the main bands related to carbonated LDH materials, as well as to Mg–Fe–OH, Fe–OH, Al–OH and Mg–Al–OH bonds [51].

The N_2_ adsorption–desorption isotherms and the pore size distribution are shown in Figure 1c–f. All catalysts present type IV(a) isotherms, with hysteresis H_2_(b)-type, which is characteristic of mesoporous materials that have a wide range of internal cavity widths. This configuration represents a complex structure with possible blocked pores. The pore size distribution curves (Figure 1c–f) revealed pore radius primarily in the mesoporous range (20–500 Å). Oxides derived from high supersaturation conditions have a narrower pore-size distribution and pore sizes are smaller than those from low supersaturation conditions; the most frequent pore radius was around 76 Å for Mg-Fe-h.s.c. and 59 Å for Mg-Al-h.s.c.

Table 1 presents the textural properties of the catalysts. Materials from the high supersaturation method (Mg-Fe-h.s.c. and Mg-Al-h.s.c.) generated oxides with a higher specific area, and smaller pore volume and pore diameter. The Mg-Al materials presented higher specific areas than Mg-Fe materials. The effect of the synthesis method was significant for both the Mg-Fe and Mg-Al oxides. Mg-Fe-h.s. and Mg-Al-h.s. catalysts from high supersaturation conditions produced LDH materials with lower crystallinity than with the low supersaturation method. This led to the formation of smaller and more organized pores in the (h.s.c.) materials, resulting in a higher specific surface area [46,52]. EDX elemental analysis results (Table 1) indicate no difference in composition between the different synthesis methods, showing similar metallic contents for all catalysts.

**Figure 2:**
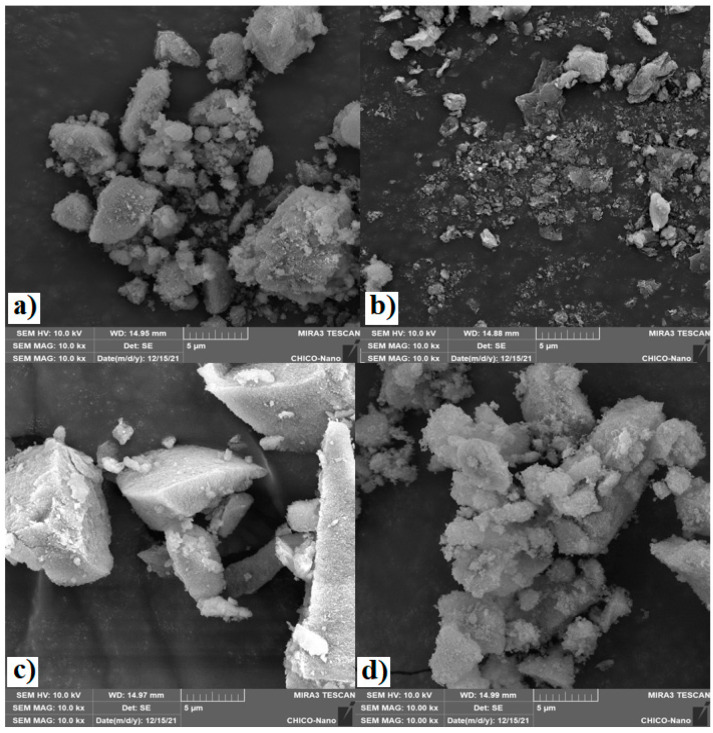
SEM images of mixed oxide catalysts: (**a**) Mg-Fe-h.s.c., (**b**) Mg-Fe-l.s.c., (**c**) Mg-Al-h.s.c. and (**d**) Mg-Al-l.s.c.

SEM images show that Mg-Fe-h.s.c. (Figure 2a) presented larger particles than the Mg-Fe-l.s.c. oxide (Figure 2b), which is formed of irregular aggregates. Very similar characteristics were observed in Mg-Al oxides, with Mg-Al-h.s.c. (Figure 2c) presenting larger particles than Mg-Al-l.s.c. (Figure 2d). The less crystalline LDH precursors obtained with the high supersaturation method produced mixed oxides with larger particle size (Figure 2a,c) and higher specific surface area (Table 1).

The low supersaturation synthesis produced more crystalline LDH; this, during calcination, resulted in smaller particles in the form of aggregates, as seen in Mg-Fe-l.s.c. (Figure 2b) and Mg-Al-l.s.c. (Figure 2d). Similarly, when we compare the Mg-Al-h.s.c. oxide with Mg-Fe-h.s.c. prepared by the same supersaturation method, the Mg-Al material presented larger particles than Mg-Fe, resulting in a higher surface area. Under that condition, the larger particles favored the higher specific surface areas, when compared to the smaller aggregated particles.

### 3.2. PET Glycolysis

PET glycolysis was performed in duplicate at 200 °C, EG:PET = 5:1 and catalyst: PET = 0.5% (Table 2). Mg-Fe-l.s.c. presented a slightly higher PET conversion than the Mg-Fe-h.s.c. In this case the material prepared by the high supersaturation method that presented a high specific area resulted in less active catalysts. SEM images show that this Mg-Fe-h.s.c. catalyst (Figure 2a) presented larger particles. A glycolysis reaction occurs when the PET macromolecules dissolved in EG are adsorbed onto the catalyst surface. In this case, it seems that the Mg-Fe-l.s.c. catalyst (Figure 2b), with agglomerates of small particles, had more accessible active surface sites able to adsorb the PET and perform the reaction. Although the Mg-Fe-l.s.c. catalyst has a smaller surface area, it has greater pore volume and pore diameter, favoring greater access of the macromolecules to their active sites and consequently greater activity. 

Similar, but more pronounced, results were observed when we compare conversion in the presence of the Mg-Al catalysts prepared from different methods. Table 2 shows that the Mg-Al-h.s.c. catalyst with large particles presented much lower PET conversion (~64.0%) than the Mg-Al-l.s.c. (~98.8%) with agglomerates of smaller particles. Catalysts from the supersaturation method presented spinel phases: Mg_0.64_Fe_2_O_4_ in the Mg-Fe-h.s.c. and MgAl_2_O_4_ in the Mg-Al-h.s.c. (Figure 1). These spinels may be responsible for the lower activity of these catalysts. Table 2 also shows that in most cases the higher the PET conversion the higher the BHET yield and monomer selectivity. This indicates that, at low conversion, the oligomer is produced first. When the conversion increases, the oligomers are depolymerized to produce more monomer. 

Concerning the influence of Fe in place of Al, the Mg-Fe-h.s.c. catalyst presented a much higher conversion (~96.8%) than the Mg-Al-h.s.c. catalyst (~64.0) (see Table 2), indicating that the influence of Fe in a catalyst that came from a similar Mg-LDH structure was significant for PET glycolysis activity. This difference was not observed for the Mg-Fe-l.s.c. and Mg-Al-l.s.c. catalysts, because the conversions were all high and not suitable for comparison of catalyst activity. These results show that Mg-Fe catalysts are very active in promoting PET glycolysis, and have the advantage of being composed of Mg and Fe biocompatible elements [29]. The use of a heterogeneous catalyst enables catalyst recovery after a reaction. However, a small quantity of catalyst may remain in the product during the recovery process or may dissolve in the very polar ethylene glycol, then partially incorporated into the final BHET product. The importance of a biocompatible catalyst is that it allows the use of BHET obtained in the repolymerization process to produce PET material suitable for food and beverage packaging applications. In the next sections we will present a detailed study of these Mg-Fe catalysts.

The activity of iron oxides in PET glycolysis has been observed by other authors. Bartolome et al. [13] attributed the phase Fe_2_O_3_ as a catalyst for PET glycolysis at 300 °C, obtaining 90% conversion. Nabid et al. [14] attributed the active sites for PET glycolysis as the Lewis acid of Fe^3+^ in Fe_2_O_3_ and basic character of N-doped graphene giving 100% conversion after 3 h. Activity of Fe_3_O_4_-B-Nitride was attributed to the Lewis acidity of Fe-oxide and basicity of boron nitride, using 10% catalyst and EG:PET~30:1 [53]. Other iron-based catalysts showed PET conversions close to 100% such as Mg-Al-O@Fe_3_O_4_ (90 min reaction) [10], ZnFe_2_O_4_ [16], Fe_2_O_3_@MoS20D-2D [17] and 2D-Fe(III) nanosheets [18]. The novel catalysts Mg-Fe of the present work reached 100% PET conversion, using less catalyst (~0.5%) and smaller EG:PET ratio (~5:1) or shorter reaction time (~1 h), indicating that our catalysts are more active than Fe-based catalysts reported in the literature. 

Several heterogeneous catalysts have been reported, demonstrating high conversions of PET and/or yielding significant amounts of BHET, as indicated in Table 3. The results obtained in this study are particularly noteworthy, as they were achieved by employing low catalyst:PET and EG:PET ratios, and shorter reaction times, and achieving high conversions of PET and high yields of BHET, especially with the Mg-Fe l.s.c. and Mg-Al l.s.c. catalysts.

**Figure 3:**
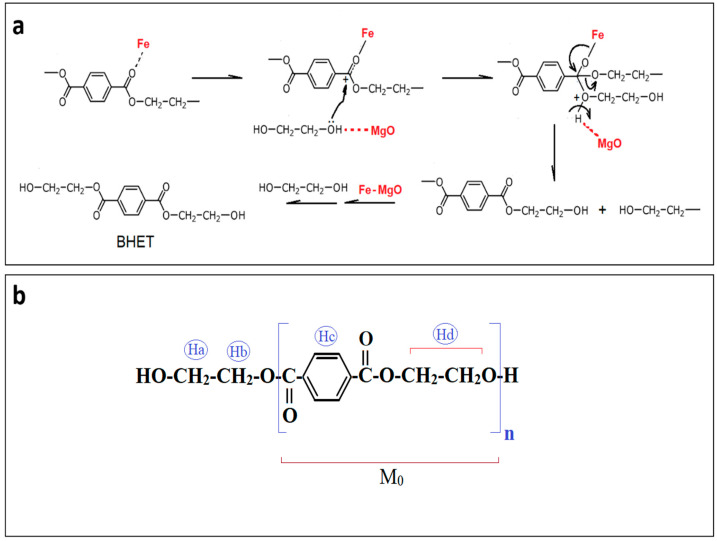
(**a**) Proposed mechanism for PET glycolysis on Mg-Fe oxide catalyst as a bifunctional catalyst. (**b**) Chemical environment of the PET molecule. Adapted from El Mejjatti et al. [54].

Based on these results, we proposed a glycolysis mechanism (Figure 3a), induced by the catalytic action of oxide catalyst, where the Fe acts as a Lewis acid that first interacts with the carbonyl group of the ester, creating a carbon site more susceptible to nucleophilic attack. The basic sites of MgO promote the nucleophilic attack of EG through the abstract of hydrogen from hydroxyl groups, then promoting a nucleophilic attack on the carbonyl, forming an intermediate containing EG, followed by the breaking of the PET chain, according to the scheme in Figure 3a.

### 3.3. Kinetics of PET Glycolysis over Mg-Fe Catalysts

**Figure 4:**
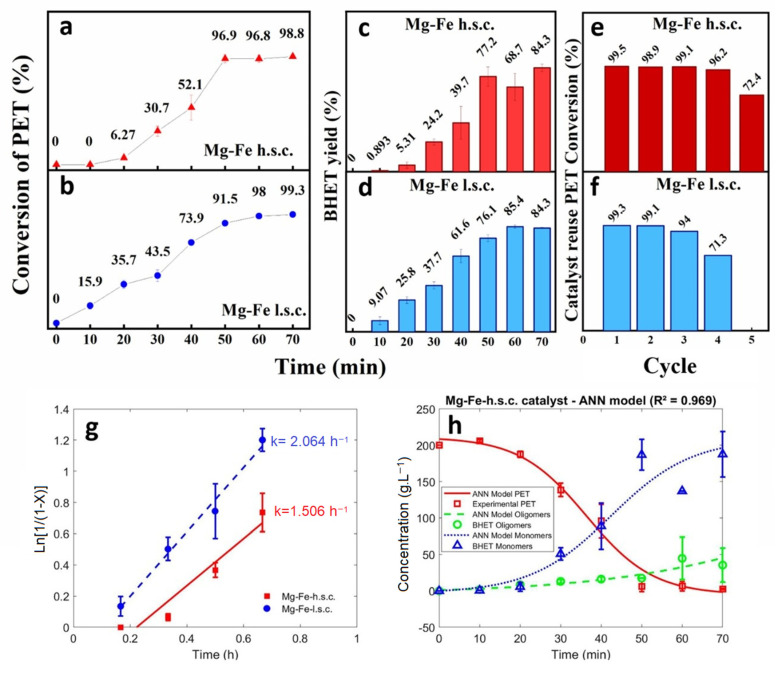
Performance of Mg-Fe-h.s.c. and Mg-Fe-l.s.c. oxide catalysts for PET glycolysis: (**a**,**b**) conversion of PET; (**c**,**d**) BHET molar yield; (**e**,**f**) catalyst reuse as conversion of PET; (**g**) linear plot of the first-order kinetic model; (**h**) neural network model and experimental data for concentrations of PET, BHET-oligomer and BHET-monomer with Mg-Fe-h.s.c. catalyst. Reaction conditions: EG:PET = 5 (g/g), catalyst:PET = 0.5% (g/g), at 200 °C and 1atm.

Once the feasibility of PET glycolysis in the presence of Mg-Fe oxides had been confirmed, two kinetic studies were performed to evaluate the influence of reaction time and to obtain a simple kinetic model. The kinetics of Mg-Fe oxides h.s.c. and l.s.c. (Figure 4) revealed different behaviors during the reactions. When we observe the kinetics of PET glycolysis (Figure 4a,b), the influence of the synthesis method on the catalyst activity is more pronounced within a shorter reaction time. For example, in a 40 min reaction, the PET conversion was 73.9% for the Mg-Fe-l.s.c. catalyst and 52.1% for the Mg-Fe-h.s.c.

The PET conversion in the presence of Mg-Fe-h.s.c. was zero in the beginning of reaction, then increased by 6% after 20 min reaction. This sigmoidal behavior is attributed to an induction time in which the EG attacked the PET chain, forming a lower molecular weight EG-insoluble polymer, resulting in low PET conversion [16]. This behavior also indicates that the reaction was limited in the initial reaction time due to an important influence of mass transfer resistance, which reduced the mass transfer of the PET dissolved in EG from accessing the active sites inside the particles [55,56]. This limitation was possibly caused by the morphology of the large particles of the catalyst (Figure 2) and low pore volume (Table 1).

Conversely, the catalyst Mg-Fe-l.s.c. presented less influence of a mass transfer resistance, with PET conversion of 34.7% after the same 20 min of reaction time (Figure 4b). In this case, from the point of view of textural analysis, the higher surface area observed for Mg-Fe-h.s. (89 m^2^·g^−1^) compared to Mg-Fe-l.s.c. (46 m^2^·g^−1^) was not decisive for the better performance of the catalyst. The lowest pore volume value of Mg-Fe-h.s.c. (0.42 cm^3^·g^−1^), in relation to that of Mg-Fe-l.s.c. (0.53 cm^3^·g^−1^), may explain the more pronounced effect of mass transfer resistance on conversion in the presence of Mg-Fe-h.s.c. due to limited access to the active sites of this catalyst. The initial first-order kinetic constant (k value obtained from the first four kinetic data) was estimated using PET conversion experimental data to the linearized model (Equation (6)), giving 1.51 h^−1^ for the Mg-Fe-h.s.c. catalyst and 2.06 h^−1^ for the Mg-Fe-l.s.c. (see Figure 4g). These values are higher than the values reported before for Fe-containing oxide catalysts [16].

As for the products, the results of the BHET yield show that, in both cases, glycolysis in the presence of Mg-Fe materials resulted in an efficient conversion of PET to BHET. The BHET yields were proportional to the reaction time and PET conversion. The maximum BHET molar yield of 74.5% was achieved with the highest conversion of 98.3%, after 70 min reaction, for the catalyst Mg-Fe-l.s.c.

A deeper look into this reaction can explain the variation between the monomer and oligomer percentages observed in the results. As proposed by the reaction mechanism in Figure 3, the PET reacts with EG molecules, producing the intermediate oligomers, which react with more EG molecules to form the BHET-monomer, according to (Equation (10)).

PET + nEG → oligomer + mEG → BHET-monomer (10)

Due to the complexity of this reaction, a neural network model (ANN) was developed to predict the concentrations of PET, BHET-oligomers and BHET-monomer as a function of reaction time. The architecture of the ANN model with one input variable and three output variables is presented in Figure S4, with estimated parameters represented by the weights and bias, after adjusting the experimental data. The calculation of each concentration (PET and BHET products) may be executed by the three-part pseudocode (input normalizing, network calculation and output denormalization), presented in Figure S5. With this code, the input reaction time is normalized from (0 to 70 min) to (−1 to +1). To obtain each normalized concentration, the model multiplies the input value to the weights connected to the neurons plus the bias values, and then calculates the value of the activation function represented by the sigmoid (Equation (11)). This value is denormalized from (−1 to +1) to (0 to 205 g L^−1^) to obtain the concentration.

(11) $$\phi (x)=\frac{2}{1+e^{-x}}-1$$

Figure 4h shows the experimental data with error bars and ANN model simulation for the concentrations of PET and BHET. These results show that, as the reaction time increased, the PET concentration decreased, and BHET-oligomers and BHET-monomer increased. This is in accordance with (Equation (10)) in which the oligomers, produced from PET glycolysis, are transformed to BHET-monomer. The ANN model was demonstrated to be able to predict the concentrations of PET and BHET with an average error of 7.4 (g L^−1^) and correlation coefficient R^2^ of 0.969.

### 3.4. Catalyst Reuse for PET Glycolysis

Both Mg-Fe materials were tested for their reusability after the PET glycolysis (Figure 4e–f). The Mg-Fe-h.s.c. performed 05 reaction cycles, and the Mg-Fe-l.s.c. performed 04 cycles. All cycles led to PET conversions above 90% and the reuse of these oxides did not show any expressive change in its conversion, until the final cycle that registered a conversion reduction around 24% for both catalysts. Although the Mg-Fe-h.s.c. catalyst presented a smaller reaction rate, it showed a higher reuse performance than the Mg-Fe-l.s.c. This can be explained by the larger particles of the Mg-Fe-h.s.c. catalyst which was less susceptible to losses by filtration during catalyst recovery and due to lower solubility in ethylene glycol. Similar recycling results have been reported with catalysts containing Fe_2_O_3_ and Fe_3_O_4_ [14,54]. 

### 3.5. Characterization of the BHET Products

The PET waste and samples of its depolymerization products were evaluated using FTIR, TG-DTG, DSC and ^1^H-NMR analyses to confirm the presence of BHET monomer after glycolysis of post-consumer PET. Figure S6 shows the infrared spectra of the post-consumer PET and BHET products. During glycolysis, the long PET chain is reduced to monomers and oligomers. Due to the similarity between the groups in the PET chains and their depolymerized glycolysis products, only small changes were observed among the samples. Primary hydroxyl groups (–OH) were identified in the bands 3608–3058 cm^−1^, referring to the increasing O–H bond of the HOCH_2_CH_2_– group added to the PET glycolysis products, which is more visible than on the PET spectra [19,56,57].

Figure S7a shows the DSC curves of PET and BHET products. The original PET sample presented a main endothermic event at 244.2 °C, which corresponds to its onset melting temperature. BHET products (oligomer and monomer) presented an endothermic event at 114.6 °C, which corresponds to the melting point of the BHET product evidencing a great difference between reactant and products. The DSC profile for BHET-oligomer and BHET-monomer were similar, indicating similar composition between them [57]. Thermogravimetric curves are shown in Figure S7b. PET sample presented a single mass loss of 80.5% between 360 and 490 °C. DTG curves of the BHET products presented a first mass loss at 176–300 °C, which corresponds to BHET degradation. The second and greater mass loss at 350–490 °C may be assigned to the PET degradation that was formed from the repolymerization of BHET at 300–350 °C, also observed in the literature [57].

^1^H-NMR spectra for the BHET obtained in the PET glycolysis in the presence of Mg-Fe-h.s.c. and Mg-Fe-l.s.c. are presented in Figures S8 and S9, respectively. Chemical shifts are observed in both spectra with a pronounced peak at δ 8.1 ppm (chemical group 1 in the structural formula of BHET), which are related to protons of aromatic rings of BHET. The protons of the methyl group (-CH_2_) closest to the ester group (-COO) and-OH are recorded between the δ signals 4.7 ppm (chemical group 2) and 3.9 ppm (chemical group 3) [56,58].

The estimated molar mass of BHET (Mn) was calculated using (Equation (12)), which is dependent on the degree of polymerization (Dpn) (Equations (13) and (14)), see Figure 3b.

(12) $$M_{n}=\mathrm{Dpn}\cdot M_{0}+M_{e}$$

(13) $$\mathrm{Dpn}=\left(I_{\mathrm{Hc}}/I_{\mathrm{Ha}}\right)$$

(14) $$M_{n}=\left(I_{\mathrm{Hc}}/I_{\mathrm{Ha}}\right)\cdot M_{0}+M_{e}$$

M_0_ is the molar mass of the repeating unit of PET (192 g·mol^−1^); the molar mass of the polymer end CH_2_CH_2_OH, M_e_ = 50 g·mol^−1^ (Figure 3b); I_Hc_ and I_Ha_ are integrals of the signals related to the aromatic protons (chemical group 1 in the structural formula of BHET) and protons close to the hydroxyl (chemical group 3), respectively [54]. The calculated molar mass was 232.7 g·mol^−1^ for BHET obtained with the Mg-Fe-h.s.c. catalyst and 231.1 g·mol^−1^ for BHET with Mg-Fe-l.s.c. These values are similar to the theoretical molar mass of BHET (~254 g·mol^−1^). These results show that the BHET obtained presented an average degree of polymerization close to ~1, indicating little presence of dimers or oligomers.

## 4. Conclusions

Mg-Fe biocompatible and non-toxic oxides were successfully prepared from layered double hydroxide precursors. These catalysts were active for the chemical recycling of PET residues via glycolysis with ethylene glycol, presenting practically 100% PET conversion, after 60 min reaction. The preparation method had significant influence on catalyst properties and activity. The high supersaturation method (without pH control) produced less crystalline oxides with larger particles and specific surface areas. The low supersaturation method (at controlled pH~10) produced smaller particles which resulted in higher PET conversion, with smaller mass transfer limitations inside their larger pore volume and pore diameter. An artificial neural network model was demonstrated to be effective to predict the concentrations of PET and BHET as a function of reaction time. The effect of Fe was evidenced by the higher activity of the Mg-Fe compared to the Mg-Al catalyst. The Mg-Fe catalysts could be recycled five times giving PET conversions up to 97%. Reaction products were confirmed as BHET monomer. Although the Mg-Fe catalyst obtained by the high supersaturation method had a slower reaction rate, it was the most effective for PET glycolysis due to multiple reuses. These novel oxide catalysts, composed of Mg and Fe biocompatible elements, are encouraging for the chemical recycling of PET residues with catalyst reuse to obtain BHET for the production of resins that can be used in food and beverage packaging.

## Figures and Tables

**Table 1:** Textural properties and chemical composition of Mg-Al and Mg-Fe oxides.

Oxide Catalyst	Surface Area ^a^ (m^2^·g^−1^)	Pore Volume ^b^ (cm^3^·g^−1^)	Average Diameter ^b^ (Å)	Mg (%)	Fe (%)	Al (%)	X ^c^
Mg-Fe-h.s.c.	89	0.42	138	41.0	59.0		0.39
Mg-Fe-l.s.c.	45	0.53	272	41.3	58.7		0.38
Mg-Al-h.s.c.	208	0.44	104	57.2		42.8	0.40
Mg-Al-l.s.c.	99	0.64	175	56.5		43.5	0.41

^a^ BET method; ^b^ BJH method; ^c^ Molar ratio M^3+^/(Mg^2+^ + M^3+^).

**Table 2:** PET conversion, BHET yield and selectivity to oligomer and monomer in the glycolysis reaction with Mg-Fe and Mg-Al oxide catalysts. Conditions: 200 °C, EG:PET = 5:1 (g/g) and catalyst: PET = 0.5% (g/g), 60 min reaction.

Catalyst	PET Conversion (%)	BHET Molar Yield (%)	BHET Oligomer Selectivity (%)	BHET Monomer Selectivity (%)
Mg-Fe-h.s.c.	96.8 ± 3.2	68.7 ± 11.0	23.6 ± 12.3	76.4 ± 12.3
Mg-Fe-l.s.c.	97.4 ± 2.6	85.4 ± 1.3	16.0 ± 5.5	84.0 ± 5.5
Mg-Al-h.s.c.	64.0 ± 10.7	52.1 ± 9.3	21.2 ± 6.0	78.8 ± 6.0
Mg-Al-l.s.c.	98.8 ± 0.1	84.0 ± 0.0	20.4 ± 4.9	79.6 ± 4.9

**Table 3:** Comparison of catalyst performance with results from other works.

Catalyst	Parameters	PET Conversion (%)	BHET Yield (%)	Reference
Mn_3_O_4_ with silica	Catalyst:PET = 1.0 wt% EG:PET = 11 300 °C, 1.3 h, 10.9 atm	-	>90%	[6]
ZnMn_2_O_4_ spinels	Catalyst:PET = 1.0 wt% EG:PET = 17.2 260 °C, 1.5 h, 5 atm	-	92.2 mol%	[7]
Ionic liquids Fe_3_O_4_@SiO_2_@(mim) [FeC_l4_]	Catalyst:PET = 15 wt% EG:PET = 0.01 180 °C, 24 h, 1 atm	-	100%	[8]
MOFs DES@ZIF-8	Catalyst:PET = 0.4% EG:PET = 5 195 °C, 0.42 h, 1 atm	100%	83.2%	[9]
Mg-Al-O@Fe_3_O_4_	Catalyst:PET = 0.5 wt% EG:PET = 5 240 °C, 1.5 h, 1 atm.	-	80 mol%	[10]
Cobalt-ferrite CoFe_2_O_4_/C10-OAC	Catalyst:PET = 2 wt% EG:PET = 5 195 °C, 2.5 h, 1 atm	100%	95.4%	[11]
γ-Fe_2_O_3_	Catalyst:PET = 0.05 wt% EG:PET = 3.3 300 °C, 1 h, 1 atm	-	90%	[13]
Fe_2_O_3_/N-graphene	Catalyst:PET = 10 wt% EG:PET = 13.3 195 °C, 3 h, 1 atm	-	100%	[14]
Fe_3_O_4_-carbon-nanotubes	Catalyst: PET = 5% ethanediol:PET = 10 190 °C, 2 h, 1 atm	-	100%	[15]
Spinel ferrites of zinc or copper ZnFe_2_O_4_	Catalyst:PET = 4% EG:PET = 5 190 °C, 6 h, 1 atm	100%	79.2%	[16]
Fe_2_O_3_@MoS_2_-2D nanocomposites	Catalyst:PET = 1.0 wt% EG:PET = 4 225 °C, 3 h, 1 atm	97%	90%	[17]
2D-Fe(III) nanosheets	Catalyst:PET = 0.01 wt% EG:PET = 18.5 200 °C, 0.5 h, 1 atm	100%	100%	[18]
MgO	Catalyst:PET = 0.25% EG:PET = 4 190 °C, 5 h, 1 atm	-	81.5%	[19]
Mg-Zn-Al (Mg–Zn)–Al	Catalyst:PET = 1.0 wt% EG:PET = 10 196 °C, 3 h, 1 atm	-	75%.	[20]
Mg-Fe h.s.c.	Catalyst:PET = 0.5 wt% EG:PET = 5 200 °C, 1 h, 1 atm	96.8%	68.7%	This work
Mg-Fe l.s.c.	Catalyst:PET = 0.5 wt% EG:PET = 5 200 °C, 1 h, 1 atm	97.4%	85.4%	This work
Mg-Al h.s.c.	Catalyst:PET = 0.5 wt% EG:PET = 5 200 °C, 1 h, 1 atm	64.0%	52.1%	This work
Mg-Al l.s.c.	Catalyst:PET = 0.5 wt% EG:PET = 5 200 °C, 1 h, 1 atm	98.8%	84.0%	This work

## Data Availability

Not applicable.

## Supplementary Materials

The following supporting information can be downloaded at: https://www.mdpi.com/article/10.3390/polym15153274/s1, Figure S1: XRD patterns of the layered double hydroxides for comparing crystallinity; Figure S2: TG and DTG curves of Mg-Fe and Mg-Al LDH materials; Figure S3: IR spectra of Mg-Al e Mg-Fe LDH; Figure S4: Architecture of the neural network model with one inlet and three outlet variables, with estimated parameters, after adjusting the experimental data; Figure S5: Computational ANN 3-part pseudocode to predict the concentrations of PET, oligomer and BHET-monomer as a function of reaction time; Figure S6: FTIR spectra of PET and main product BHET monomer; Figure S7: (a) DSC thermal analysis curves of PET and BHET oligomer (P1) and monomer (P2); and (b) Derivative thermogravimetric curves of PET and BHET oligomer (P1) and monomer (P2), under nitrogen; Figure S8: ^1^H-NMR spectra of BHET obtained in the presence of Mg-Fe-h.s.c catalyst; Figure S9: ^1^H-NMR spectra of BHET obtained in the presence of Mg-Fe-l.s.c catalyst; Table S1: Lattice parameters and crystallite size for Mg-Fe and Mg-Al LDH materials; Table S2: Mass losses from the thermogravimetric analyses of Mg-Fe and Mg-Al LDH materials.
