# Supplementary material for: Chemical Recycling of PET Using Catalysts from Layered Double Hydroxides: Effect of Synthesis Method and Mg-Fe Biocompatible Metals
Source: Polymers (Basel). 2023 Aug 2;15(15):3274. doi: 10.3390/polym15153274 (PMC10422272; doi:10.3390/polym15153274)
Supplement: Supplementary file 1 [file polymers-15-03274-s001.zip › polymers-2506615-supplementary.pdf]

# Chemical Recycling of PET Using Catalysts from Layered Double Hydroxides: Effect of Synthesis Method and Mg-Fe Biocompatible Metals

**Ana P. Arcanjo [1], Denisson O. Liborio [1], Santiago Arias [1], Florival R. Carvalho [2], Josivan P. Silva [1,3], Bernardo D. Ribeiro [4], Marcos L. Dias [5], Aline M. Castro [6], Roger Fréty [1], Celmy M. B. M. Barbosa [1,*] and Jose Geraldo A. Pacheco [1,*]**

[1] Laboratory of Refining and Cleaner Technology (LabRefino-Lateclim), Department of Chemical Engineering, Institute for Petroleum and Energy Research (i-LITPEG), Federal University of Pernambuco, Recife 50740-550, PE, Brazil; ariashenao@gmail.com (S.A.)
[2] Fuel Laboratory, Department of Chemical Engineering, Institute for Petroleum and Energy Research (i-LITPEG), Federal University of Pernambuco, Recife 50740-550, PE, Brazil
[3] Engineering and Technology Center, Uninassau University, Paulista 53401-440, PE, Brazil
[4] Biochemical Engineering Department, School of Chemistry, Federal University of Rio de Janeiro, Rio de Janeiro 21941-909, RJ, Brazil
[5] Macromolecules Institute, Federal University of Rio de Janeiro, Rio de Janeiro 21941-598, RJ, Brazil
[6] Research, Development and Innovation Center (Cenpes), Petrobras, Rio de Janeiro 21941-915, RJ, Brazil
* Correspondence: celmy@ufpe.br (C.M.B.M.B.); jose.pacheco@ufpe.br (J.G.A.P.)

## SUPPLEMENTARY MATERIAL

### 3. Results and discussion

*3.1 Characterization of the catalyst*

**Table S1.** Lattice parameters and crystallite size for Mg-Fe and Mg-Al LDH materials

| LDH sample | 2θ (003) | $d_{003}$ (Å) | *c* (Å) | 2θ (110) | *a* (Å) | Crystallite Size[a]. (nm) |
|---|---|---|---|---|---|---|
| Mg-Fe-h.s. | 11.35 | 7.80 | 23.39 | 59.29 | 3.12 | 12.87 |
| Mg-Fe-l.s. | 11.35 | 7.80 | 23.39 | 59.29 | 3.12 | 13.70 |
| Mg-Al-h.s. | 11.28 | 7.85 | 23.54 | 60.40 | 3.07 | 12.06 |
| Mg-Al-l.s. | 11.39 | 7.77 | 23.31 | 60.40 | 3.07 | 14.25 |

[a]Crystallite size from Scherrer´s Equation

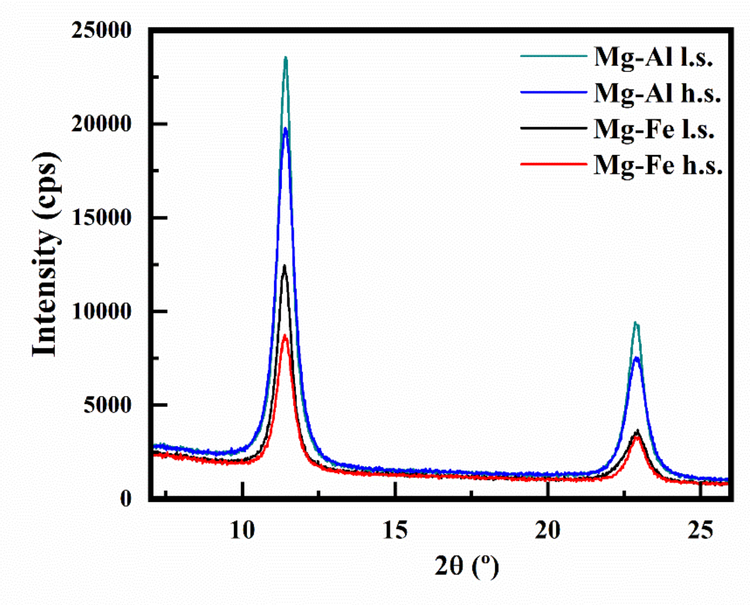


**Figure S1.** XRD patterns of the layered double hydroxides for comparing crystallinity.

Figure S2 shows the TG-DTG curves of LDH materials. Thermal decomposition followed three events: dehydration from adsorbed and interlayer water; dehydroxylation and decarboxylation (ARIAS et al. 2018). Table S2 shows the mass losses of each temperature range. A closer look at the mass loss in the first event shows that the degree of hydration in materials synthesized at high supersaturation is greater than at low supersaturation. The second mass loss dehydroxylation was ~12.7 % for all materials. The decarboxylation stage occurred in one step for the (h.s.) materials with about 25 % mass loss; while for the (l.s.) materials, additional small losses were detected at 370°C, that may be from the release of a residual carbonate (KOWALIK et al. 2013). Another fact observed was that there was a small delay between the thermal events for Mg-Al when compared to the Mg-Fe materials. The higher crystallinity observed for Mg-Al samples on the XRD patterns could be evidence of the formation of a material that is more stable structurally and more resistant to the thermal decomposition.

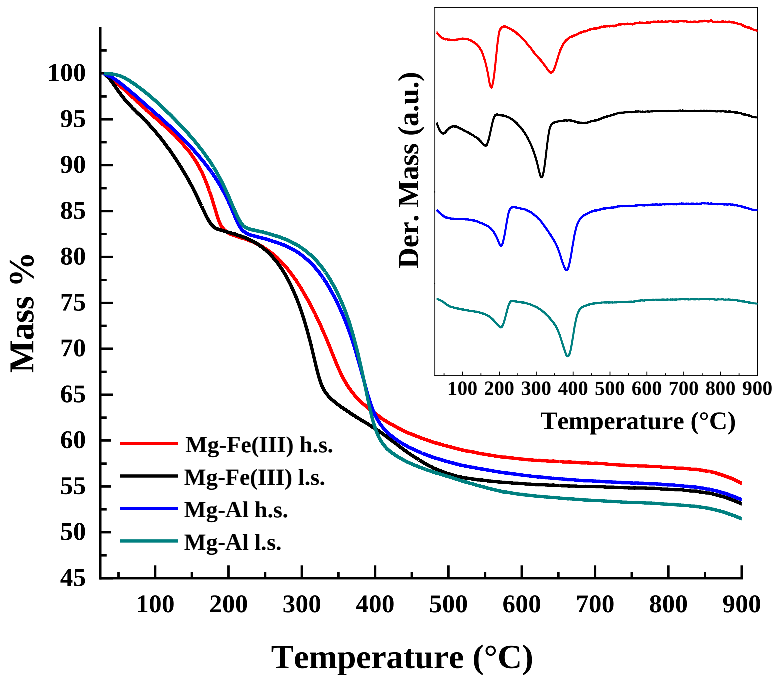


**Figure S2.** TG and DTG curves of Mg-Fe and Mg-Al LDH materials.

**Table S2.** Mass losses from the thermogravimetric analyses of Mg-Fe and Mg-Al LDH materials.

| LDH sample | Event range (°C) (mass loss %) | | | | Total mass loss (%) |
|---|---|---|---|---|---|
| Mg-Fe-h.s. | 25-107°C (5.4 %) | 107-212°C (12.4 %) | 212-649°C (24.3 %) | - | 42.1 % |
| Mg-Fe-l.s. | 25-76°C (4.2 %) | 76-192°C (13.0 %) | 192-366°C (19.7 %) | 366-610°C (7.8 %) | 44.7 % |
| Mg-Al-h.s. | 25-114°C (5.3 %) | 114-234°C (12.4 %) | 234-613°C (26.1 %) | - | 43.8 % |
| Mg-Al-l.s | 25-116°C (4.1 %) | 116-232°C (13.0 %) | 232-486°C (26.5 %) | 486-585°C (2.0 %) | 45.6 % |

The FTIR spectra of LDH materials are plotted in Figure S3. A band in 3700–3300 $cm^{-1}$ is assigned to (vOH) related to hydroxyl and interlayer $H_2O$. The bending mode of the water (δ H2O) is around 1600 $cm^{-1}$. The band in 1300 $cm^{-1}$ is due to asymmetric stretching of the v3 mode of $CO_3^{2-}$. Bands between 700–600 $cm^{-1}$ may be related to the stretching of Mg–Fe–OH, Fe–OH, Al–OH, and Mg–Al–OH bonds (ELHALIL et al. 2016).

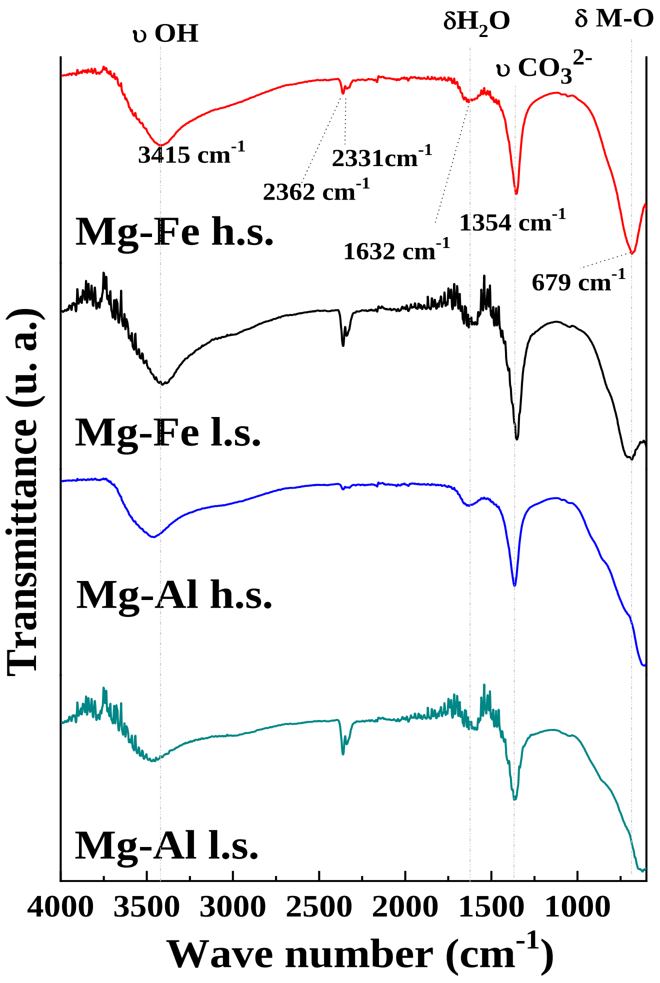


**Figure S3.** IR spectra of Mg-Al e Mg-Fe LDH.

**3.2 PET glycolysis**

*3.3 Kinetics of PET Glycolysis over Mg-Fe Catalysts*

The Figure S4 illustrates the architecture of the neural network generated after training. In this network, time has been normalized between -1 and 1, which corresponds to the time interval of 0 to 70 minutes. The output consists of the concentrations of PET, oligomers, and monomers, represented on a scale from -1 to 1, which need to be converted to the range 0 to 205.89 g·L$^{-1}$.

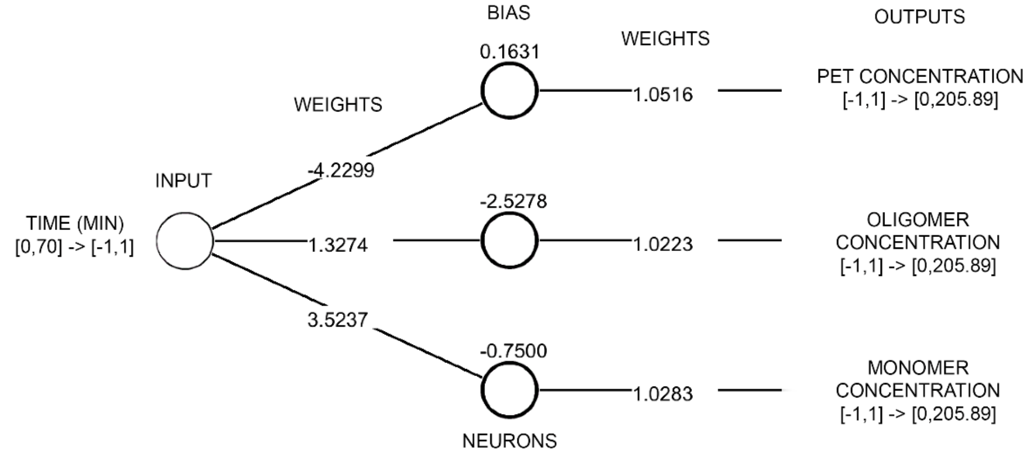


**Figure S4.** Architecture of the neural network model with one inlet and three outlet variables, with estimated parameters, after adjusting the experimental data.

```
//Normalization
Input(i) = 2 * Time/ 70 - 1;
//Calculation
Output(1) = 1.051553*(2/(1+exp(-1*(-4.229918* Input (1)+0.1630613)))-1); //PET
Output (2) = 1.022267*(2/(1+exp(-1*(1.327412* Input (1)-2.527785)))-1); //Oligomer
Output (3) = 1.028256*(2/(1+exp(-1*(3.523658* Input (1)-0.7499706)))-1); //Monomer
//Denormalization
Output (i) = 102.945* Output (i)+102.945;
```

**Figure S5.** Computational ANN 3-part pseudocode to predict the concentrations of PET, oligomer and BHET-monomer as a function of reaction time.

*3.5. Characterization of the BHET products*

The PET waste and samples of its depolymerization products were evaluated by FTIR, TG-DTG, DSC, and $^1$H-NMR analyses to confirm the presence of BHET monomer after glycolysis of post-consume PET. Figure S6 shows the infrared spectra of the post-consume PET and BHET products. During glycolysis, the long PET chain is reduced to monomers and oligomers. Due to the similarity between the groups in the PET chains and their depolymerized glycolysis products, only small changes were observed among the samples. Primary hydroxyl groups (–OH) were identified on the bands 3608–3058 cm$^{-1}$, referring to the increasing O–H bond of the $HOCH_2CH_2$– group added to the PET glycolysis products, which is more visible than on the PET spectra. Methyl group bonds (-CH-) are recognized at 2957 and 2872 cm$^{-1}$ (CAPELETTI et al. 2017; CHEN, 2003). The aryl group is seen at 1500 cm$^{-1}$; the symmetrical deformation form of the C–O–O bond related to esters linked to unsaturated structures on the bands at 1261 and 1118 cm$^{-1}$, and the stretching related to the carbonyl group (C=O) of unsaturated esters at 1717 cm$^{-1}$. The

other are very similar in shape and intensity to the initial PET, with the benzene p-substitution at 892 and 727 $cm^{-1}$ (ALZUHAIRI et al. 2018).

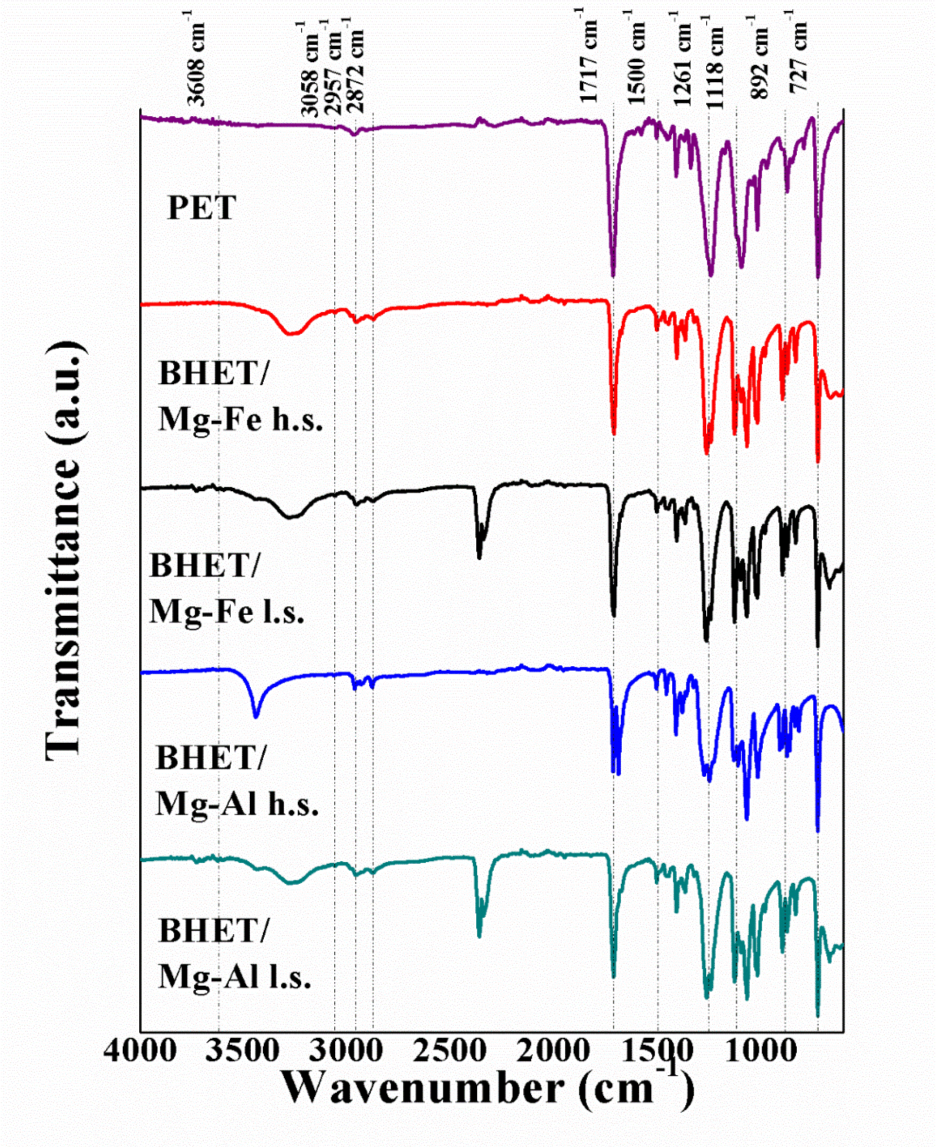


**Figure S6.** FTIR spectra of PET and main product BHET monomer.

Figure S7a shows the DSC curves of PET and BHET products: oligomer (P1) and monomer (P2). The original PET sample presented a main endothermic event at 244.2 °C, which corresponds to its onset melting temperature. BHET products (oligomer and monomer) presented an endothermic event at 114.6 °C, which correspond to the melting point of BHET product evidencing a great difference between reactant and products. In general, the DSC profile for BHET-oligomer (P1) and BHET-monomer (P2) were similar, indicating similar composition between them (CHEN, 2003). Thermogravimetric curves are shown in Figure S7b. PET sample

presented a single mass loss of 80.5 % between 360–490 °C. DTG curves of the BHET products presented a first mass loss in 176–300 °C, which corresponds to the BHET degradation. The second and greater mass loss in 350–490 °C may be assigned to the PET degradation that was formed from the repolymerization of BHET at 300–350 °C, also observed in the literature (CHEN, 2003).

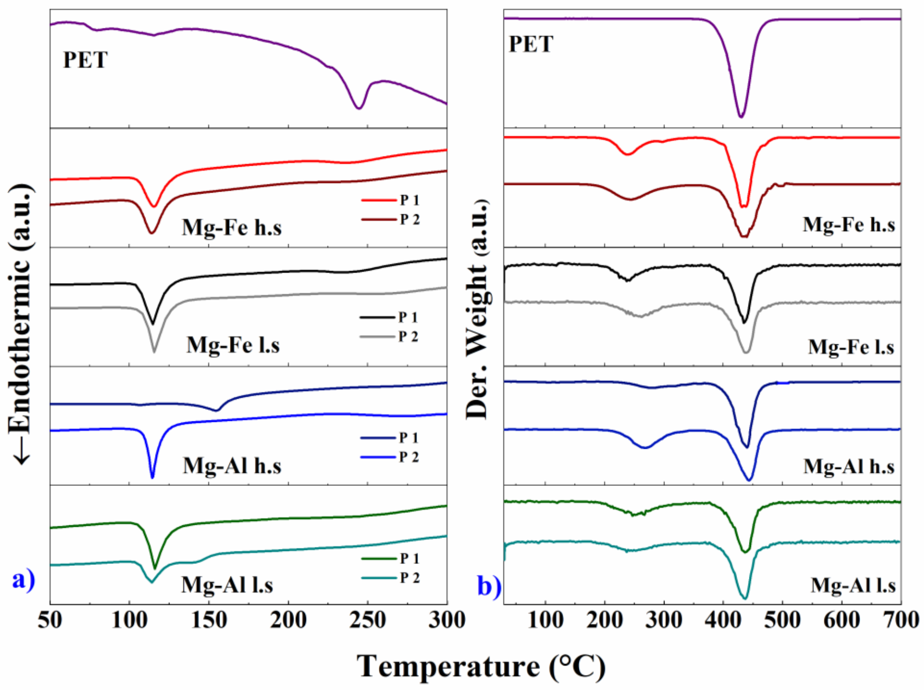


**Figure S7.** a) DSC thermal analysis curves of PET and BHET oligomer (P1) and monomer (P2); and b) Derivative thermogravimetric curves of PET and BHET oligomer (P1) and monomer (P2), under nitrogen.

$^{1}$H-NMR spectra for the BHET obtained in the PET glycolysis in the presence of Mg-Fe-h.s.c. and Mg-Fe-l.s.c. are presented in Figures S8 and S9, respectively. Chemical shifts are observed in both spectra with a pronounced peak at δ 8.1 ppm (chemical group 1 in the structural formula of BHET), which are related to protons of aromatic rings of BHET. The protons of the methyl group (-$CH_2$) closest to the ester group (-COO) and-OH are recorded between the δ signals 4.7 ppm (chemical group 2) and 3.9 ppm (chemical group 3), respectively; these results agree with the literature (NICA et al. 2015). The other two peaks recorded represent the presence between the samples of the solvent CDCl3 (δ 7.1 ppm) used to record the $^{1}$H-NMR spectra and water (δ 1.8 ppm) (CAPELETTI et al. 2017).

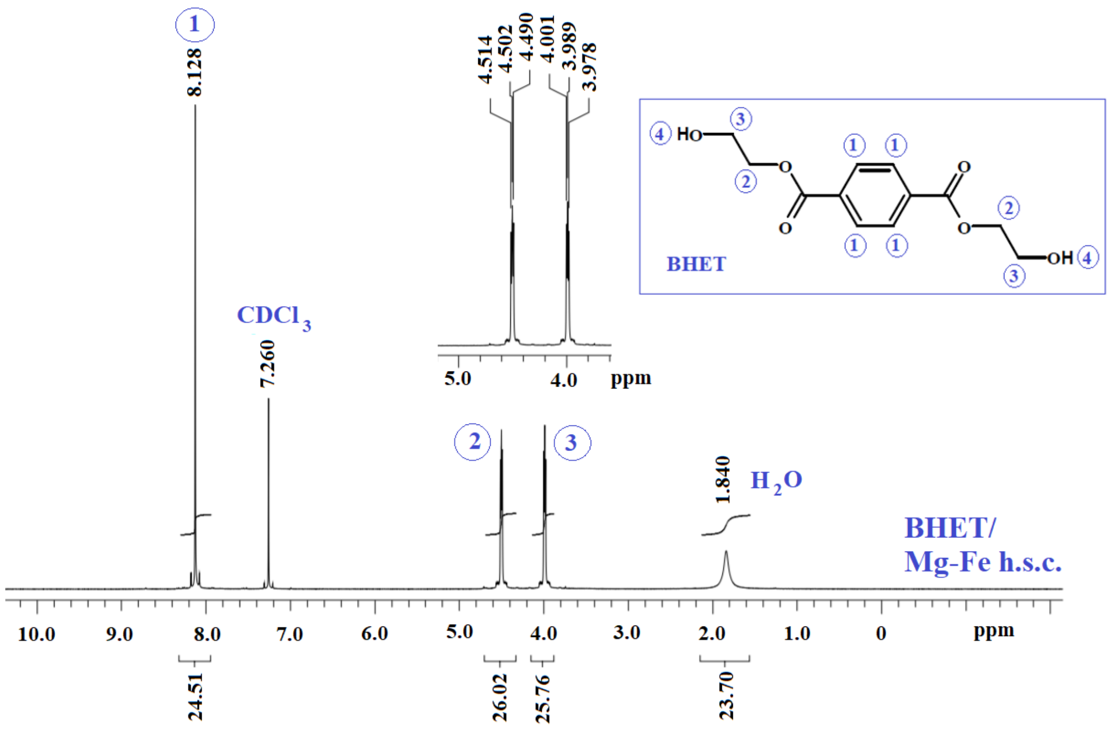


**Figure S8.** $^{1}$H-NMR spectra of BHET obtained in the presence of Mg-Fe-h.s.c catalyst.

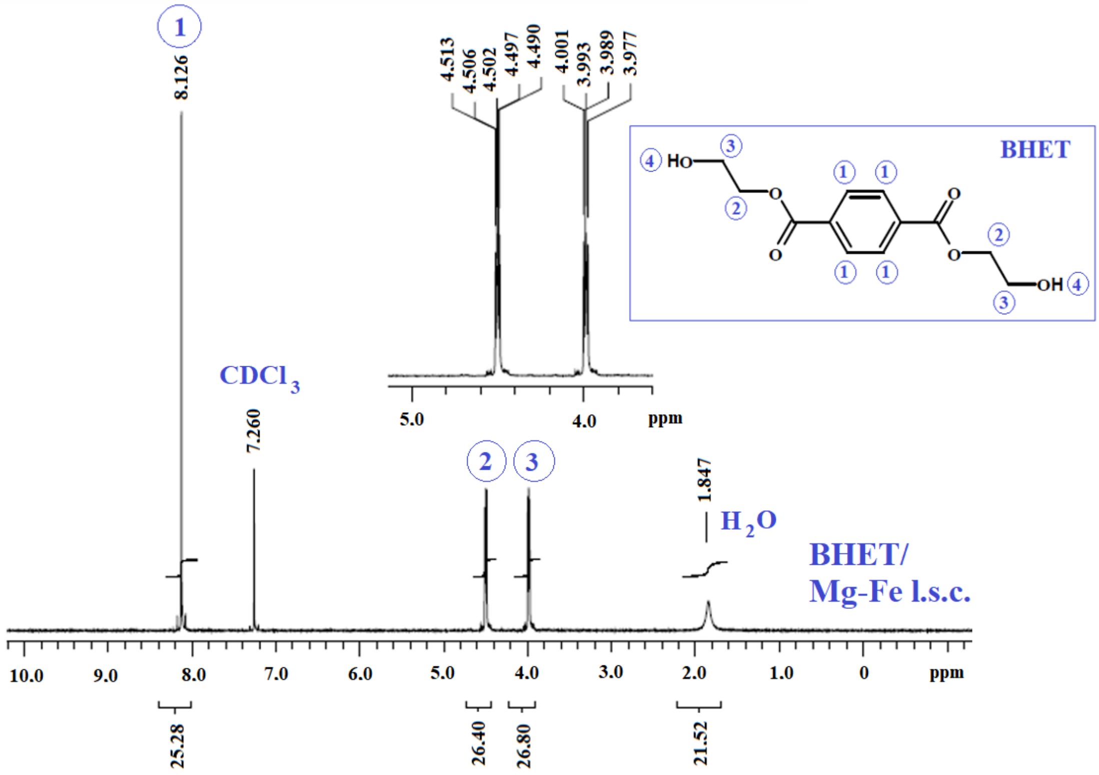


**Figure S9.** $^{1}$H-NMR spectra of BHET obtained in the presence of Mg-Fe-l.s.c catalyst.

## References


Alzuhairi, M.A.A.; Khalil, B.I.; Hadi, R.S. Nano MgO catalyst for chemical depolymerization of polyethylene terephthalate (PET). *Iraq. J. Phys.* **2018**, 16, 85–93. https://doi.org/10.30723/ijp.v16i36.33.

Arias, S.; Sousa, L.V.; Barbosa, C.B.M.; Silva, A.O.S.; Fréty, R.; Pacheco, J.G.A.Preparation of NiAlZr-terephthalate LDHs with high Al and Zr content and their mixed oxides for cyclohexane dehydrogenation. *Appl. Clay Sci.* **2018**, *166*, 137–145. https://doi.org/10.1016/j.clay.2018.09.020.

Capeletti, M.R.; Passamonti, F.J.; Optimization of reaction parameters in the conversion of PET to produce BHET. *Polym. Eng. Sci.* **2017**, 58, 1500–1507. https://doi.org/10.1002/pen.24720.

Chen, C.H. Study of Glycolysis of Poly (ethylene terephthalate) Recycled from Postconsumer Soft-Drink Bottles. III. Further Investigation. *J. Appl. Polym. Sci.* **2003**, *87*, 2004–2010. https://doi.org/10.1002/app.11694.

Elhalil, A.; Qourzal, S.; Mahjoubi, F.Z.; Elmoubarki, R.; Farnane, M.; Tounsadi, H.; Sadiq, M.; Abdennouri, M.; Barka, N. Defluoridation of groundwater by calcined Mg/Al layered double hydroxide. *Emerg. Contam.* **2016**, *2*, 42–48. https://doi.org/10.1016/j.emcon.2016.03.002.

Kowalik, P.; Konkol, M.; Kondracka, M.; Próchniak, W.; Bicki, R.; Wiercioch, P. Memory effect of the CuZnAl-LDH derived catalyst precursor—In situ XRD studies. *Appl. Catal. A* **2013**, *464*–465, 339–347. https://doi.org/10.1016/j.apcata.2013.05.048.

Nica, S.; Hanganu, A.; Tanase, A.; Duldner, M.; Iancu, S.; Draghici, C.; Filip, P.I.; Bartha, E. Glycolytic Depolymerization of Polyethylene Terephtalate (PET) Wastes Organic vs. metal-catalysis. *Rev. Chim. Bucharest* **2015**, *66*, 1105–1111.
